# Unilateral syphilitic optic neuritis without meningitis or uveitis

**DOI:** 10.1002/ccr3.5678

**Published:** 2022-04-20

**Authors:** Shun Yamashita, Masaki Tago, Tomoyo M. Nishi, Shu‐ichi Yamashita

**Affiliations:** ^1^ 469771 Department of General Medicine Saga University Hospital Saga Japan

**Keywords:** impaired visual acuity, magnetic resonance imaging, rapid plasma regain, syphilitic optic neuritis, treponema pallidum hemagglutination assay

## Abstract

A 69‐year‐old man with left eye pain and visual impairment was diagnosed with syphilitic optic neuritis, who was successfully treated by penicillin. Although it is difficult to decide syphilis as the direct cause of optic neuritis, it is essential to diagnose syphilis in every patient with optic neuritis.

## Graphical abstract

It is difficult to decide whether syphilis is the cause of optic neuritis. All patients with optic neuritis should receive serological tests for syphilis, and patients with positive results should be administered with penicillin G prior to steroid.

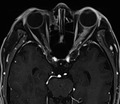

A 69‐year‐old man had left eye pain 5 days previously with subsequent deterioration of his visual acuity (VA), which became finger counting at 10 cm with impaired left direct light reflex and relative afferent pupillary defect on admission. Fundoscopy examination showed left optic disk edema (Figure 1). The results of serum rapid plasma reagin (RPR) and treponema pallidum hemagglutination assay (TPHA) were positive. Cerebrospinal fluid showed negative RPR and TPHA without pleocytosis. T1‐weighted orbital contrast‐enhanced magnetic resonance imaging with fat suppression showed a swollen enhanced lesion in the left optic nerve (Figure 2). Twenty‐four million units per day of penicillin G (PCG) were administered for 2 weeks for probable diagnosis of syphilitic optic neuritis (SON) with consequent recovery of his VA to 50/100 in 4 months.

**FIGURE 1:**
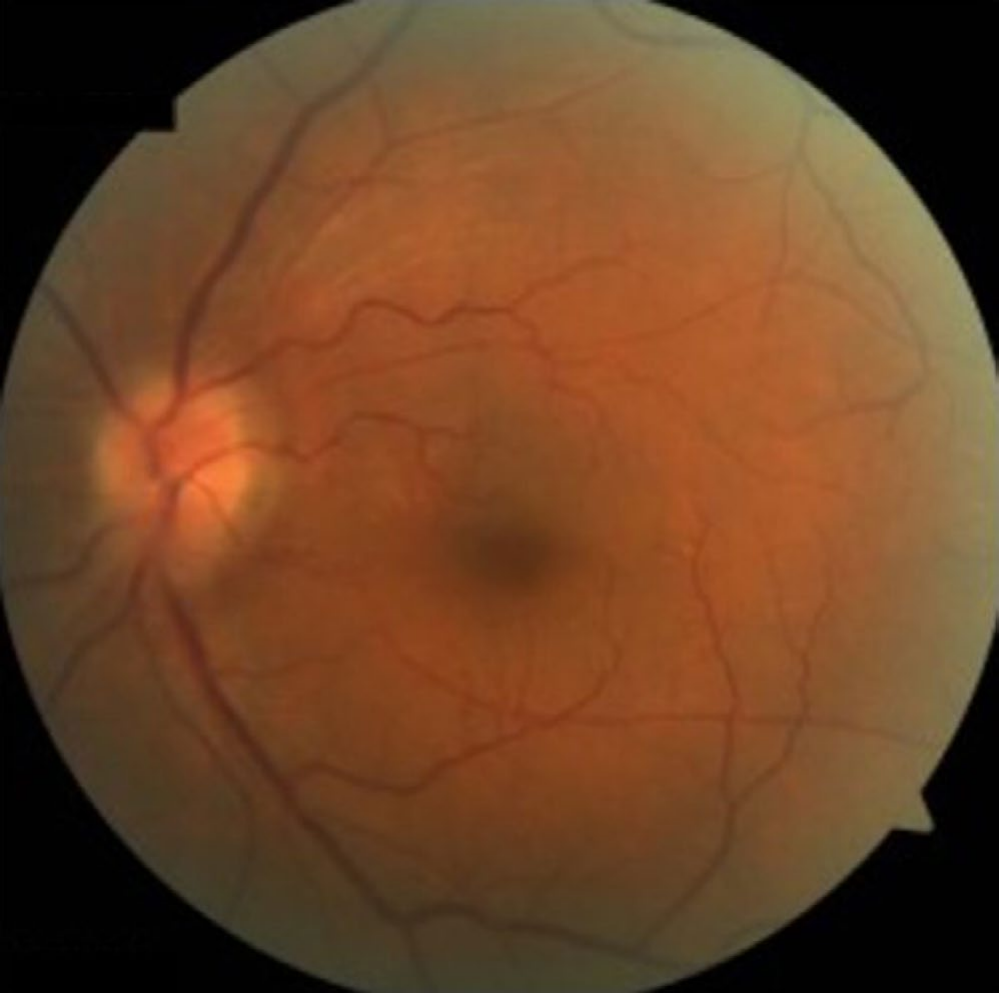
Findings of left funduscopic examination on admission. The left optic disk is poorly marginated, showing the findings of left optic disk edema

**FIGURE 2:**
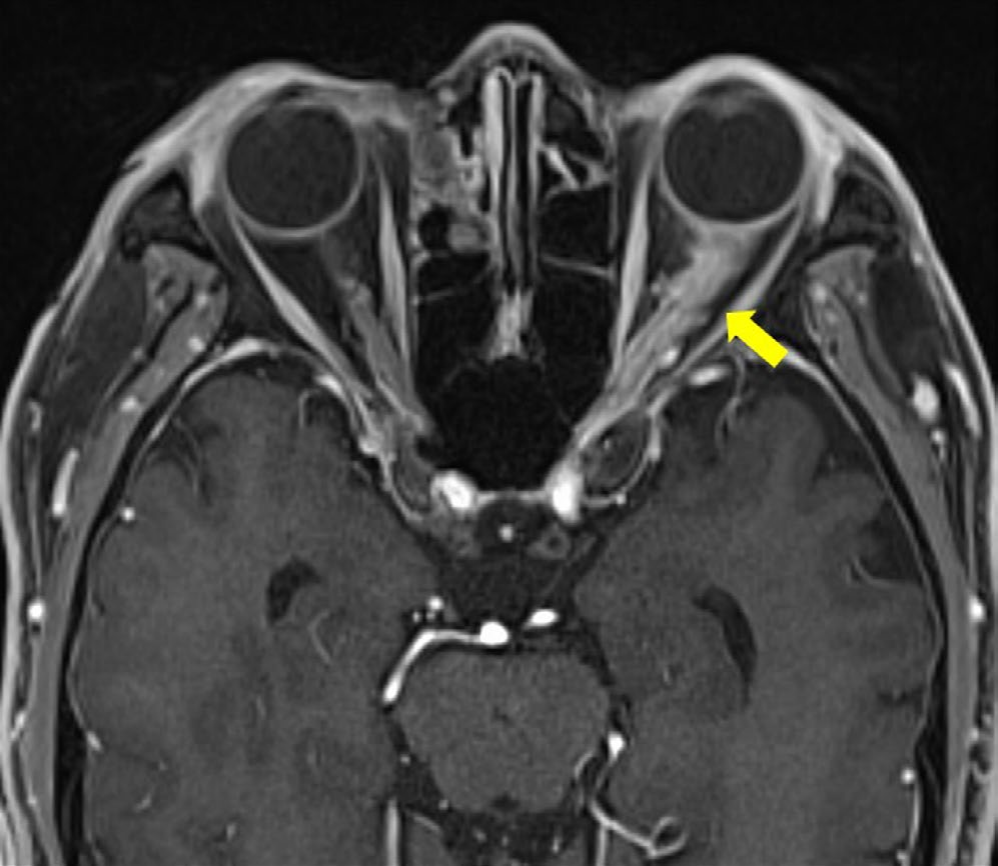
Findings of T1‐weighted orbital magnetic resonance imaging with contrast enhancement and fat suppression on admission. T1‐weighted orbital imaging with contrast enhancement and fat suppression using volumetric interpolated breath‐hold examination sequences shows swollen enhanced lesion in the left optic nerve (arrow)

SON is a rare type of neurosyphilis.^1^ Although it is desirable to distinguish SON from noninfectious optic neuritis because steroid without PCG can exacerbate SON,^2^ such differentiation is difficult without complicated syphilitic meningitis. Our patient was consequently diagnosed with SON, due to recovery of his VA after PCG therapy. It is essential to perform serological tests for syphilis in every patient with optic neuritis, which makes it possible to initially treat SON with PCG.

## CONFLICTS OF INTEREST

The authors state that they have no Conflict of Interest (COI).

## AUTHOR CONTRIBUTIONS

SY involved in concept, literature search, and drafting of manuscript. MT and TMN involved in concept and literature search. SI‐Y involved in concept and revision of manuscript.

## ETHICAL APPROVAL

This manuscript conforms to the provisions of the Declaration of Helsinki in 1995 (as revised in Brazil 2013).

## CONSENT

Written informed consent was obtained from the patient to publish this report in accordance with the journal's patient consent policy.

## Data Availability

The data that support the findings of this study are available from the corresponding author upon reasonable request.
